# Green Tea Polyphenols Modulated Cerebral SOD Expression and Endoplasmic Reticulum Stress in Cardiac Arrest/Cardiopulmonary Resuscitation Rats

**DOI:** 10.1155/2020/5080832

**Published:** 2020-02-25

**Authors:** Wanxiang Hu, Huihui Wang, Quan Shu, Menghua Chen, Lu Xie

**Affiliations:** ^1^Department of Physiology, School of Pre-Clinical Sciences, Guangxi Medical University, Nanning, Guangxi, China; ^2^Qilu Medical University, Zibo, Shandong, China; ^3^Hubei University of Science and Technology, Xianning, Hubei, China; ^4^Institute of Cardiovascular Diseases, The Second Hospital Affiliated to Guangxi Medical University, Nanning, Guangxi, China

## Abstract

**Background:**

Reducing cerebral ischemia-reperfusion injury is crucial for improving survival and neurologic outcomes after cardiac arrest/cardiopulmonary resuscitation (CA/CPR). The purpose of this study is to investigate the neuroprotective effects of green tea polyphenols (GTPs) concern with the modulation of endogenous antioxidation and endoplasmic reticulum stress.

**Methods:**

After subjecting to CA/CPR, rats were randomized into the saline group (NS, *n* = 40) and the GTPs group (GTPs, *n* = 40) and the GTPs group (GTPs, *n* = 40) and the GTPs group (GTPs,

**Results:**

Comparing with that in NS group, GTPs increased the expression of SOD1 and SOD2 at 12 h, 24 h, 48 h, 72 h, and the expression of GRP78 at 24 h and 48 h (*p* < 0.05) butdecreased caspase-12, CHOP, caspase-3 level, and apoptotic number of neurons (*p* < 0.05) butdecreased caspase-12, CHOP, caspase-3 level, and apoptotic number of neurons (

**Conclusion:**

GTPs exert neuroprotective effects via mechanisms that may be related to the enhancement of endogenous antioxidant capacity and inhibition of endoplasmic reticulum stress in CA/CPR rat models.

## 1. Introduction

Cardiac arrest is a common and intractable condition frequently encountered in hospitals and outside of hospitals [1]. Although more rapid and effective resuscitation is thought to alleviate the outcomes after cardio arrest (CA), the mortality and morbidity after CA/CPR remain high; that is due to ischemia-reperfusion injury occurring in organs in the body, especially in the brain [2–4]. Cerebral ischemia-reperfusion injury (CIRI) is a very complicated cascade of reactions involving multiple mechanisms, such as oxidative stress, calcium overload, mitochondrial dysfunction, inflammatory reaction, and neuronal apoptosis [5–7]. Current researches have indicated that oxidative stress plays a vital role in the pathogenesis of CIRI with a characteristic of the imbalance of oxidative and antioxidant defense system [8, 9]. When the redox balance is disturbed, accumulated free radicals trigger cell membrane lipid peroxidation and can also initiate neuronal apoptotic cascades through mitochondria, endoplasmic reticulum, or death receptors, resulting in neural dysfunction and cell death [10–12].

In recent years, endoplasmic reticulum (ER) stress has attracted widespread attention as a new mechanism of apoptosis, which is related to the pathological process of cerebral ischemia. After the ER stress, initiating the unfolded protein response (UPR) will help to restore the activity of endoplasmic reticulum and participate in the coordination of nerve cell apoptosis so as to improve neurological disorders [13, 14]. Furthermore, one study reported that dexmedetomidine can partially inhibit ER stress-induced apoptosis through modulating ER stress-related protein (for example, inducing GRP78 expression but inhibiting CHOP, caspase-3, and phosphorylated-JNK expression), thereby attenuating brain damage after ischemia-reperfusion [15]. Similarly, the inhibition of the expression of ER stress apoptotic proteins such as caspase-12, CHOP, Bax/Bcl-2, caspase-9, and caspase-3 may exert protective effect via PI3K/AKT signaling pathway on ER stress-induced neuronal apoptosis in in vivo or in vitro experiments [16]. The above evidence interests us to find out a probability against CIRI concerning ER stress modulation.

GTP is a phenolic hydroxyl compound extracted from tea leaves and has strong antioxidant capacity [17–20]. Studies have shown that the active constituents of green tea polyphenols, the phenolic hydroxyl group of epigallocatechin-3-gallate (EGCG), can bind to hydrogen radicals, lipid peroxides and free iron to block the oxidative chain reaction, inhibiting apoptosis induced by focal cerebral ischemia and hypoxia reperfusion [9]. It has been reported that EGCF can attenuate ER stress-induced apoptosis of tubular epithelial cells in kidney, accompanied by decreased ER stress-related markers [21]. Our previous studies showed that GTPs can reduce the content of reactive oxygen species and malondialdehyde (MDA) in brain tissue of CPR rats, enhance SOD activity, significantly improve the score of neurological deficit, and prolong the survival time after resuscitation [22]. In addition to enhancing the activity of SOD, GTPs can increase the expression of SOD and inhibit neuronal apoptosis through modulating ER stress remains elusive. Therefore, in this study, we used the typical cardiac arrest (CA)/CPR model to confirm antioxidant effects of GTPs treatment through detecting SOD1, SOD2 level and to reveal the effect of GTPs on ER stress and apoptosis by detecting the apoptosis indices as well as ER stress-associated marker proteins such as GRP78, caspase-12, and CHOP.

## 2. Materials and Methods

### 2.1. Experimental Animals and Protocol

The experimental rats were treated according to the Guidelines for the Care and Use of Laboratory Animals and the study protocol was approved by the animal ethics committee of Guangxi Medical University. Ninety healthy male Sprague-Dawley rats, aged 6 to 8 weeks, were purchased from the Animal Experimental Center of Guangxi Medical University (Nanning, Guangxi). All rats were fed with normal rat chow and water ad libitum and kept on a 12 h light and dark cycles. Rats were allowed to acclimatize for 1 wk and then randomly divided into 3 groups: 40 rats in each group of the NS group and the GTPs group, and 10 rats in the Sham group. The present protocol referenced to our previous study in grouping and GTPs doses as follows [22]. Rats in the NS group and the GTPs group were randomly divided into four subgroups at four time points: 12 h, 24 h, 48 h and 72 h. In addition to the Sham group, the other groups of rats were induced into CA by esophageal electrical stimulation and then CPR. Rats were administered immediately after resuscitation via femoral vein in double-blind by 0.9% saline (1 ml/kg), GTPs (10 mg/kg), respectively. At various time points, experimental rats were killed by intraperitoneal injection of excess sodium pentobarbital and brain tissues were harvested according to different experimental requirements, in which half of them were fixed in 4% buffered paraformaldehyde and made into paraffin sections for immunofluorescence staining and TUNEL staining. Another five rats' cortices in each group were collected and homogenized for Western blotting. The Sham group underwent surgical operations, such as the femoral artery, vein and tracheal intubation, and monitoring of ECG and blood pressure, without inducing CPR.

### 2.2. CA/Cerebral Ischemia-Reperfusion Modeling

The CA/cerebral ischemia-reperfusion model of rats was established as previously described [23]. Briefly, rats were anesthetized with an intraperitoneal injection of 2% sodium pentobarbital (3 ml/kg) and fixed in the supine position. Tracheal intubation and surgical incision in the left inguinal region were performed separately, and cardiac rhythm was monitored using a standard II lead electrocardiogram. Then, two 20-gauge catheters filling with sodium heparin saline (5 IU/ml) were inserted into the left femoral artery or vein for monitoring hemodynamic and delivering drugs, respectively. The pressure sensor was connected to a 4-channel physiological recorder (BL-420E New Century software; Chengdu Tai Meng Technology&Market Co., Ltd., China). After all physiological indicators were observed for several minutes, the rats were induced CA by a pacing electrode (alternating current, 12V) placed in the esophagus. Cardiac arrest was defined as a loss of aortic pulsation or aortic pulse pressure less than 10 mmHg, and mean arterial pressure (MAP) less than 20 mmHg together with ventricular fibrillation, pulseless electrical activity, or cardiac arrest. Seven minutes after CA, CPR was initiated with a volume-controlled small animal ventilator (DH-150; Department of Medical Instruments, Zhejiang University, China), chest compression, and adrenaline (0.02 mg/kg, IV). The manual chest compression speed is 180 beats per minute following a metronome. Restoration of spontaneous circulation (ROSC) was defined as an unassisted pulse with a mean arterial pressure value not lower than 50 mmHg for maintaining 1 minute or longer.

### 2.3. Immunofluorescence Staining of SOD1, SOD2, CHOP, and Caspase-3

The prepared paraffin slices were roasted, dewaxed and citrated buffer high pressure repaired, then they were naturally cooled, removed endogenous peroxidase by adding 3% hydrogen peroxide, and incubated with different primary antibody (SOD1, ab13498, 1 : 200, Abcam, USA; SOD2, ab13534, 1 : 200, Abcam, USA; CHOP, #2895, 1 : 200, CST, USA; caspase-3, ab32351, Abcam, USA; 1 : 200) in a wet box at 4°C overnight. The negative control group was replaced with PBS instead of the primary antibody. Washing in PBS three times for 5 minutes each time, secondary antibody (ALexar Fluor647, ab150079, 1 : 300, Abcam, USA) was incubated in a cassette for 1 h at 37°C, and then washed with PBS again. At last, Nuclear was counterstained with DAPI for 2 min and rinsed 3 times by PBS. The appropriate amount of antifluorescence-attenuating sealer (Solarbio, Shanghai, China) was used for sealing. Five different fields of fluorescence were observed under a conjugated double-focus microscope (Nikon, Japan). The fluorescence density was analyzed by Image-Pro Plus 6.0 software.

### 2.4. TUNEL and DAPI Staining

The apoptotic neurons were detected by TUNEL staining using a commercial kit (116848817910, Roche, USA) according to the manufacturers' instructions. Paraffin sections of 5 *μ*m thickness from tissues were deparaffinized and rehydrated conventionally, then rinsed twice in PBS for 5 min and incubated with Proteinase K working solution for 30 min at room temperature. After washing in PBS again, the labeling reaction was performed using 50 *μ*L TUNEL reagent for each sample, except negative control in which reagent without enzyme, incubated for 1 h at 37°C. Following washing, the sections were treated with the terminal deoxynucleotidyl transferase (TdT) reaction. Finally, the paraffin sections were counterstained with DAPI for 2 min. All images were examined and captured by the confocal microscope (Nikon AI-Japan). TUNEL-positive apoptotic cells exhibiting green fluorescent granules were counted in five randomly selected microscopic fields at a microscopic magnification of 200x.

### 2.5. Western Blotting

The expression of ES stress-related proteins was detected by Western blot. Protein concentration was assayed by BCA reagents. 80 *μ*g protein samples from each sample were electrophoresed in 10% gels. Then the separated proteins were transferred onto a polyvinylidene difluoride (PVDF) membrane (Bio-Rad, Hercules, CA, United States). 5% skimmed milk was used to block the nonspecific binding proteins for 40 min at room temperature, and then the membranes were incubated overnight at 4°C with the following primary antibodies: GRP78 (1 : 1000, Cell Signaling Technologies, United States), caspase-12 (1 : 1000, Abcam, United States), and tubulin (1 : 1000 Cell Signaling Technologies, United States). Subsequently, the membranes were washed thrice with TBST and the membranes were incubated with secondary antibodies rabbit polyclonal antibody (1 : 1000, Abcam, United States) for 60 min. Finally, An enhanced Chemiluminescence was used to visualize the bands, which were captured on X-ray film. The relative intensity of the bands was analyzed by Image-Pro Plus 6.0 software, and the band densities of target proteins were normalized to that of tubulin.

### 2.6. Statistical Analysis

The obtained data were analyzed using SPSS17.0 software. All values were presented as the means ± standard deviation (SD). Significant differences between groups are shown by one-way analysis of variance and the results are analyzed by software with *p* < 0.05 considered statistically significant.

## 3. Results

### 3.1. GTPs Enhanced Fluorescence Density of SOD1 and SOD2

**Figure 1:**
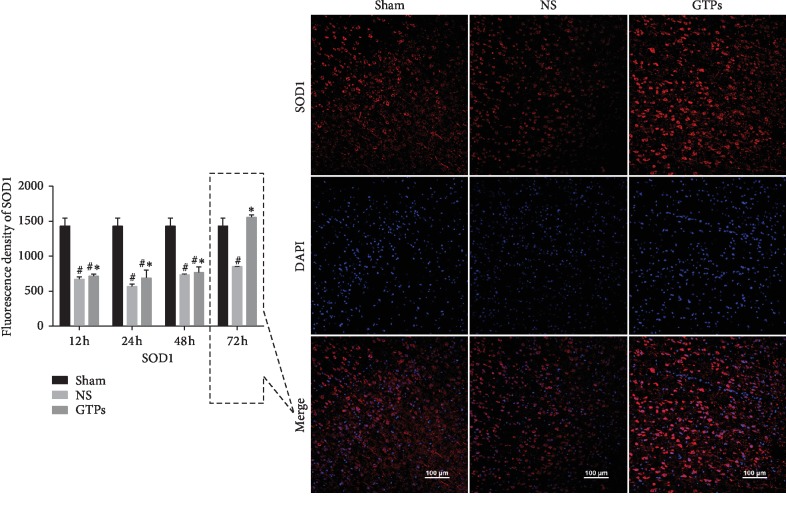
GTPs increased SOD1 expression. The left bar shows the fluorescence density of SOD1 in different groups at various time points. *p* < 0.05 vs. Sham group. ^*∗*^*p* < 0.05 vs. NS group. Especially, at 72 h, the fluorescence of the TP group is obviously stronger than that of the model group and Sham group, and the Sham group is stronger than the model group.

**Figure 2:**
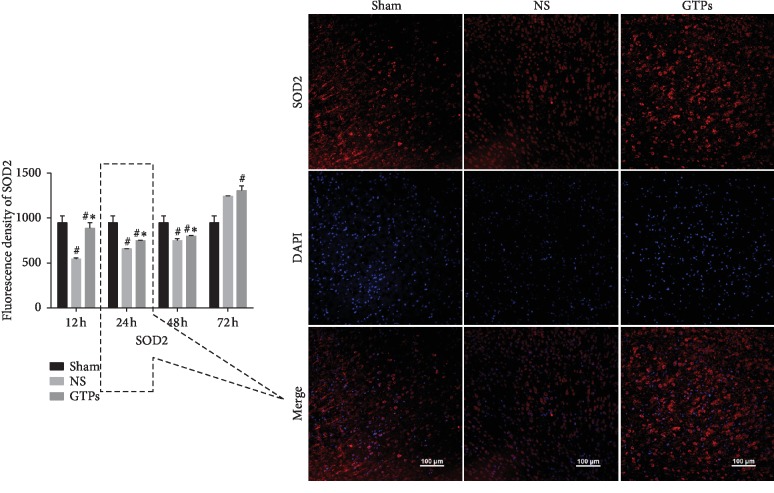
GTPs increased SOD2 expression. The left bar shows fluorescence density of SOD2 contrast in different groups at various time points. ^#^*p* < 0.05 vs. Sham group. ^*∗*^*p* < 0.05 vs. NS group. At 12 h and 24 h, the fluorescence of the TP group was obviously stronger than that of the model group, and the model group is stronger than the Sham group. Here, we show the SOD2 fluorescent staining picture at 24 h.

The fluorescent staining of SOD1 and SOD2 was, respectively, shown in Figure 1 and Figure 2. We choose the representative picture at a special time point to show the most significant change (see Figure 1 (right) and Figure 2 (right)). As Figure 1 (left) and Figure 2 (left) showed, the fluorescence density of SOD1 in the NS group was significantly decreased at each time point (*p* < 0.05) and SOD2 decreased at 12 h, 24 h, and 48 h compared with the Sham group. Whereas in the GTPs group, both SOD1 and SOD2 were higher than the NS group at all time points. The above results show that GTPs exert an antioxidative role through enhancing the expression of SOD1 and SOD2 in the process of CIRI after CA/CPR. Furthermore, SOD1 and SOD2 may exert their own effects at different times, respectively.

### 3.2. GTPs Reduced Neuronal Apoptosis

**Figure 3:**
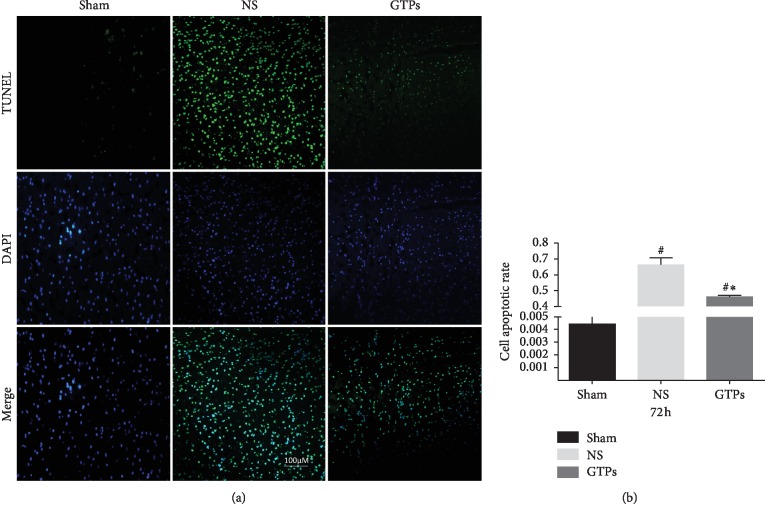
Apoptotic rate at 72 h after ROSC. (a) Representative photomicrographs of triphosphate nick-end labeling (TUNEL) staining in the three groups. 4′, 6-diamidino-2-phenylindole (DAPI, blue) was used to counterstained cell nuclei (magnification × 200). Scale bar = 100 *μ*m. (b) The apoptosis rate was acquired by the ratio of TUNEL-stained cells to the total cells of DAPI staining. ^#^*p* < 0.05 compared with the Sham group; ^*∗*^*p* < 0.05 compared with the NS group.

The neuronal apoptosis was detected by TUNEL staining. DNA damage was detected and morphology of normal nuclei was detected by DAPI staining. The apoptosis rate is expressed as the ratio of TUNEL-stained cells to the number of DAPI staining cells. Comparing apoptosis rates at various time points, we found that only at 72 h after ROSC, the expression of TUNEL-positive cells in the NS group and the GTPs group was significantly higher than that in the Sham group, and the number of TUNEL-positive cells was significantly lower than that in the NS group (Figure 3, *p* < 0.05) after GTP_S_ treatment. At least, GTP_S_ can effectively reduce neuronal apoptosis caused by cerebral ischemia-reperfusion injury in CA/CPR rats model.

### 3.3. GTPs Reduced Caspase-3 Expression

**Figure 4:**
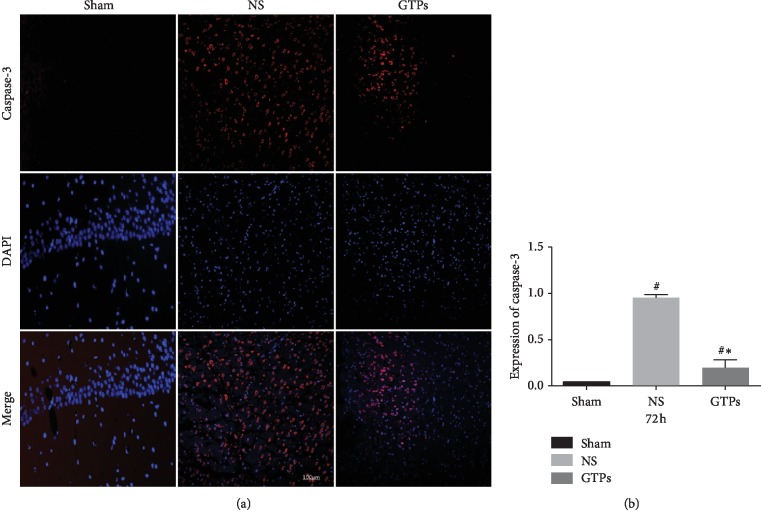
Effects of GTPs on caspase-3 expression at 72 hours after CPR (200x). (a) Fluorescence density of caspase-3 at 72 h. Red fluorescence represented the positive expression of caspase-3, and the blue fluorescence was the normal nucleus. (b) The quantification of the positive expression of caspase-3. Values were represented as mean Th, *n* = 5 for each group. ^#^*p* < 0.05 compared with the Sham group; ^*∗*^*p* < 0.05 compared with the NS group.

Caspase-3 was a proapoptotic protein in the classic apoptotic pathway. We chose it to measure the apoptosis in this experiment.  Figure 4 showed that the expression of caspase-3 in the GTPs group and NS group was higher than that in the Sham group at 72 h, but the expression level of caspase-3 in the GTPs group was lower than that in the NS group (*p* < 0.05). The experimental data at 12, 24, and 48 h time points showed no significant difference by statistical analysis. These results suggest that GTPs effectively alleviated the expression of caspase-3, which indicated the antiapoptosis role of GTPs during the pathological process of CIRI.

### 3.4. Effect of GTPs on the Expression of Caspase-12 and GPR78

**Figure 5:**
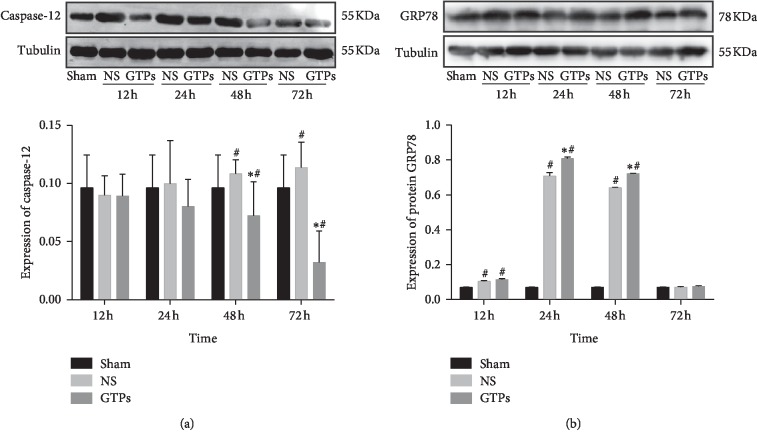
Effects of GTPs treatment on the protein levels of caspase-12 and GRP78 in different groups at different time points after ROSC. (a) Western blotting bands of caspase-12 and the relative expression of caspase-12. (b) Western blotting bands of GRP78 and the relative protein expression of GRP78. Values are represented as mean, *n* = 5 for each group. ^#^*p* < 0.05, compared to the Sham group, ^*∗*^*p* < 0.05 indicate comparison with the NS group.

To confirm that the ER stress participated in the neuroprotective role of GTP_S_ against CIRI, caspase-12 and GPR78 protein expression were detected in the brain tissues with or without GTPs treatment. As shown in Figure 5(a), we found no significant difference in the expression of caspase-12 between the 12th and 24th hour. The expression of caspase-12 protein in the NS group was higher than that in the Sham group at 48 h and 72 h (*p* < 0.05), while the expression of caspase-12 protein in the GTPs group was lower than that in the NS group and Sham group (*p* < 0.05).

As Figure 5(b) showed, the expression of GRP78 in the NS group began to increase at 12 h after CPR and reached the peak at 24 h (*p* < 0.05). The most significant increase of GRP78 expression occurred in the NS group and GTPs group at 24 h and 48 h (*p* < 0.05), and compared with the NS group, the GTPs group also increased significantly (*p* < 0.05). There was no statistical difference between groups at 72 h. So, GPR78 exerts the role in ER stress mainly at 24–48 hours after resuscitation.

Our data indicated that GRP78, as a vital regulator of ER stress, significantly changed after GTPs treatment and the expressions of caspase-12 and GPR78 were different at various periods.

### 3.5. CHOP Expression in the Cerebral Cortex

**Figure 6:**
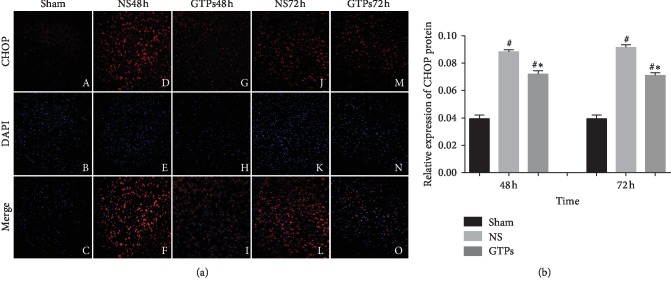
GTPs attenuated CHOP levels induced in CA/CPR rats. (a) Cerebral cortex fluorescence staining in rats at 48 hours and 72 hours. Images were captured by objective lens ×200. Red (a, d, g, j, m) indicated CHOP positive cell expression in the cerebral cortex. Blue (b, e, h, k, n) stained by DAPI (4′, 6-diamidino-2-phenylindole) represented all cells. (c, f, i, l, o) showed merging pictures. (b) The relative expression of CHOP protein. Data are shown as mean ± standard deviation, *n* = 5. ^#^*p* < 0.05 compared with the Sham group; ^*∗*^*p* < 0.05 compared with the NS group.

Figure 6 showed that immunofluorescence staining expression of CHOP in the brain of the NS group (NS48Hd, e and NS72Hj, k) and GTPs group (GTPs48Hg, h and GTPs72Hm, n) was significantly increased compared with the Sham group (*p* < 0.05) and reached a peak at 72 h after CPR. GTPs decreased significantly the expression of CHOP compared with the NS group (*p* < 0.05), but in the early stage, for example, the expression of CHOP at 12 h or 24 h was not statistically significant.

The experimental results of CHOP were consistent with the expression of caspase-12. After early self-repair and self-balance, if it cannot rescue the injury caused by CIR, in the later stage after CIRI, excessive endoplasmic reticulum stress may eventually promote brain cells to undergo apoptosis and cause irreversible damage. Therefore, early treatment intervention cannot be overemphasized.

## 4. Discussion

The present study expanded our previous research and investigated the potential neuroprotective mechanisms of GTPs concerning endogenous antioxidation and ER stress. The imbalance of oxidation and antioxidation is a direct reason for deficits of neurological functions and high mortality during CIRI [24, 25]. GTPs possess higher antioxidant activity than vitamin E and vitamin C [26, 27]. Our team and other researchers have reported that GTPs can improve CIRI outcomes in local cerebral ischemia-reperfusion and CPR models, relating to its effects of increasing SOD activity and decreasing ROS, MDA production [9, 28]. At present, this study further confirmed that GTPs can produce endogenous antioxidant effects by promoting the expression of SOD1 (CuZn-SOD, mainly located in the cytoplasm) and SOD2 (Mn-SOD, mainly located in mitochondria) in the brain.

Similarly, our previous research has demonstrated that GTPs improved the morphology of brain cells and reduced cell death after CPR by H&E staining. In order to observe the antiapoptotic effect of GTPs, we used typical TUNEL staining here, and the results are consistent with previous experimental results. Interestingly, we also found that GTPs modulating excessive oxidative stress contributed to an antiapoptotic effect. After GTPs treatment, we observed the ratio of apoptosis cells and caspase-3 expression both decreased while the expression of SOD1 and SOD2 both increased, indicating that there is a potential relationship between antioxidation and antiapoptotic function of GTPs. More SOD expression by GTPs means less apoptosis in the brain under certain circumstances.

To elucidate the additional mechanism of GTPs on antiapoptosis apart from antioxidation, we investigated the possibility of GTPs on the ER pathway concerning apoptosis in cerebral injury post-CA/PCR, since it is known that ER stress relates to pathophysiology of ischemia-reperfusion [29, 30]. As the important site for protein folding and synthesis, ER is very sensitive to the homeostasis unbalance of the body [31]. In ischemia and hypoxia, oxidative stress disturbs the ER homeostasis leading to the accumulation of misfolded or unfolded protein, triggering unfolded protein response (UPR) [32]. The protein chaperone GRP78 is an important regulator of ER stress, apoptosis, and cell survival. Under physiological conditions, GRP78 usually combines with three ER transmembrane proteins, respectively, such as double-stranded RNA-dependent protein kinase-like endoplasmic reticulum kinase (PERK), activating transcription factor 6 (ATF6) and inositol requiring enzyme-1 (IRE-1), thus maintaining their inactive state. Once the accumulation of misfolded or unfolded protein occurs in cells induced by various pathophysiological factors, GRP78 dissociates with these three proteins and initiated ER stress. PERK, ATF6, and IRE-1 can promote the refolding of new proteins and removes misfolded and unfolded proteins to restore ER homeostasis via respective pathways, playing an adaptive reaction favoring cell survival, whereas in hyperactive ER stress, the three proteins push the reaction to apoptosis by upregulating C/EBP-homologous protein (CHOP) and caspase-12 expression. CHOP promotes proapoptotic bak and Bax expression, as well as inhibiting antiapoptotic Bcl-2 expression. caspase-12, an important member of the caspase-originating inflammatory family, is the only murine protein that exists in the endoplasmic reticulum. Activated caspase-12 can be transported into the cytoplasm to bind to caspase-9 and then activate caspase-3 [10]. Both CHOP and caspase-12 lead to the classic apoptotic pathway.

Our present results showed a significant increase of brain GRP78 level in rats at 12–48 h post-CA/PCR, especially at 24–48 h, CHOP and caspase-12 also increased at 24–48 h. However, in comparison to NS treatment, GTP treatment increased GRP78 expression further, while decreased CHOP and caspase-12 expression. We considered that GTPs modulating ER stress are more favorable to eliminate misfolded and unfolded proteins resulting in adaptive reaction than intriguing CHOP and caspase-12 expression inducing apoptosis, followed by reduced caspase-3 expression and apoptotic rate. Coinciding with our study, other evidence also suggested that the increased expression of GRP78 induced in ER stress exerted cytoprotection against hypoxia-induced cell death [33]. Furthermore, in in vivo experiment, the overexpressed GRP78 exerted cardioprotective effects and mitigates cell apoptosis by suppressing ROS after myocardial I/R injury [34]. Inhibition of caspase-12 pathway can reduce neuronal apoptosis in cerebral ischemia, and caspase-12-deficient rats can resist ER stress-induced apoptosis, while other death stimuli can still induce apoptosis [29, 30, 35]. So, our study suggests that the modulation of ER stress could be potential access for alleviating brain cell death after CA/PCR.

Certainly, there are limitations existing in our current research. Our experiments did merely a preliminary research of GTPs on potential antiapoptotic mechanisms concerning ER stress and endogenous antioxidation. In further studies, ER stress agonist and inhibitor, and more antioxidant proteins need to undergo study for evaluating GTPs effects.

## 5. Conclusion

Our study reveals that decreased SOD expression in brain tissue after CPR implies downregulation of endogenous antioxidant capacity and dysfunction of endoplasmic reticulum stress and cell apoptosis which all may be pathological factors of CIRI. After GTPs treatment, both apoptosis-related indicators and endoplasmic reticulum stress-related proteins are reduced. We demonstrate that GTPs inhibit neuronal apoptosis involved with the increase of cerebral endogenous antioxidative ability and modulation of ER stress, which provides additional information about GTPs' effective mechanism on cerebral protection against CIRI after CA/CPR.

## Data Availability

All data and models used during the study are available from the corresponding author upon request.
